# Heterogeneous expression of ARID1A in colorectal cancer indicates distinguish immune landscape and efficacy of immunotherapy

**DOI:** 10.1007/s12672-024-00955-9

**Published:** 2024-03-31

**Authors:** Xin Guan, Luying Cui, Yuli Ruan, Lin Fang, Tianjiao Dang, Yanqiao Zhang, Chao Liu

**Affiliations:** 1 Department of Gastrointestinal Medical Oncology, Harbin Medical University Cancer Hospital, 150 Haping road, Harbin, Heilongjiang 150001 People’s Republic of China; 2 Key Laboratory of Tumor Immunology in Heilongjiang, Harbin, China; 3 Clinical Research Center for Colorectal Cancer in Heilongjiang, Harbin, China; 4 Phase I Clinical Research Center, The Affiliated Hospital of Qingdao University, Qingdao, 266003 China

**Keywords:** Colorectal cancer, ARID1A, Heterogeneous expression, CD8, Immunotherapy

## Abstract

**Objective:**

AT-rich interaction domain 1A (ARID1A) mutant tumors show active anti-tumor immune response, which is the potential indication of immunotherapy. However, the relationship between the heterogeneous ARID1A expression and the immune response and immunotherapy efficacy in colorectal cancer (CRC) is still unclear.

**Methods:**

We collected 1113 cases of patients with stage I-IV CRC who underwent primary resection at Harbin Medical University Cancer Hospital. ARID1A expression in CRC tissues was assessed via immunohistochemistry (IHC). CD8, CD163 and FOXP3 were stained by IHC to identify the immune landscape. Clinicopathological features of patients were compared using statistical tests like the Wilcoxon-Mann–Whitney test or χ2 tests. Kaplan–Meier survival analysis with log-rank tests were employed.

**Results:**

Heterogeneous ARID1A expression was categorized into integrity expression, complete expression deficiency (cd-ARID1A), partial expression deficiency (pd-ARID1A), and clonal expression deficiency (cld-ARID1A). ARID1A-deficient expression was significant association with dMMR (P value < 0.001). Patients with ARID1A deficiency, compared to ARID1A-proficient patients, exhibited increased infiltration levels of CD8 + P value < 0.0001), CD163 + P value < 0.001), and FOXP3 + P value < 0.001).cells within the tumor tissue. However, in different subgroups, only samples with complete or partial deficiency of ARID1A showed a higher abundance of lymphocyte infiltration. In patients with ARID1A-clonal expression deficiency tumor, the infiltration patterns of three immune cell types were comparable to those in ARID1A-proficient patients. Heterogeneous ARID1A expression is related to the different prognosis and immunotherapythe efficacy in CRC patients.

**Conclusion:**

Heterogeneous ARID1A expression is accompanied by a different immune landscape. CRC patients with ARID1A-clonal expression deficiency do not benefit from the treatment of immune checkpoint inhibitors (ICIs).

## Introduction

AT-rich interaction domain 1A (ARID1A) functions as a subunit of the Switch/Sucrose Non-Fermentable (SWI/SNF) chromatin remodeling complex and ranks among the most frequently mutated genes in colorectal cancer (CRC) (9.3–11.1%) [1–3]. The majority of ARID1A mutations are inactivating nonsense or frame-shift mutations, ultimately leading to the loss of ARID1A protein expression [4]. Generally, ARID1A has been identified as a specific tumor suppressor in CRC, and variations of ARID1A have been reported to be correlated the tumorigenesis and the poor prognosis of CRC [5, 6].

In addition to its roles in tumorigenesis and gene expression, the role of ARID1A in regulation of tumor immune response has been proposed. A recent study found that ARID1A recruited MSH2 to chromatin and facilitated mismatch repair [7]. Additionally, ARID1A has been validated to interact with checkpoint kinase ATR to regulate the efficient repair of DNA double-strand breaks (DSBs) [8–10]. ARID1A deficiency impaired DNA damage repair (DDR), resulting in increased mutagenesis in tumor cells and activation of the adaptive immune response [11]. Meanwhile, ARID1A-deficient increased tumor programmed cell death receptor ligand 1(PD-L1) expression through activation of phosphatidylinositol 3-kinase (PI3K)/protein kinase B (PKB) signaling. Given the impact of ARID1A deficiency on the immune response, ARID1A has been proven a promising biomarker for immunotherapy in multiple cancers. Encouragingly, in microsatellite stability (MSS) CRC patients, ARID1A mutation is defined as an immunologically active subgroup with abundant intra-tumoral T-cell infiltration, and these patients have the potential to benefit from immunotherapy, such as programmed death-1 monoclonal antibody (PD-1 mAb) [7, 12, 13].

Although the ARID1A-deleted subset of CRC has demonstrated promising immunotherapy value, the effect in clinical practice is indeed unsatisfactory. Some patients with ARID1A mutations do not benefit from immune checkpoint inhibitors (ICIs) therapy [14]. Chou A. et al. reported that heterogeneous expression of ARID1A in CRC [15]. However, whether the heterogeneous expression of ARID1A is accompanied with different immune landscape is not clear. Here, we report the relationships between heterogeneous expression of ARID1A and different immune landscapes and immunotherapy effects in CRC.

## Methods and materials

### Patients and endpoints

pMMR CRC who underwent primary resection at Harbin Medical University Cancer Hospital (HMUCH; Harbin, China) between January 4, 2013, and December 30, 2015, were retrospectively reviewed in this study. Patients with additional tumors, those who received preoperative therapy, or individuals lacking complete histopathological data were excluded from the analysis. The endpoint overall survival(OS) was defined as the initial diagnosis date to the last follow-up date; the latter was defined as the date of death or the last live follow-up during this study.

### Immunohistochemistry (IHC)

In the study, we analyzed the expression of ARID1A and other biomarkers in tissue samples from 1113 colorectal cancer patients, including 1109 with adenocarcinoma and 4 with adenosquamous carcinoma. The study utilized primary tumor paraffin sections that were 4 μm thick for IHC analysis. The process involved steps such as roasting, deparaffinization, and rehydration, followed by antigen retrieval with EDTA buffer and inactivation of peroxidase activity with 3% H2O2. Antibodies against ARID1A (1:500, ab182560, Abcam, Cambridge, UK), CD8 (1:500, ab101500, Abcam), CD163 (1:500, ab182422, Abcam) and FOXP3 (1:500, ab200334, Abcam) were used for incubation overnight at 4 ℃. All the above antibodies are rabbit monoclonal antibodies. The DAB IHC Detection Kit along with biotinylated secondary antibodies were employed, and samples were counterstained with Mayer’s Hematoxylin solution.

**Fig. 1:**
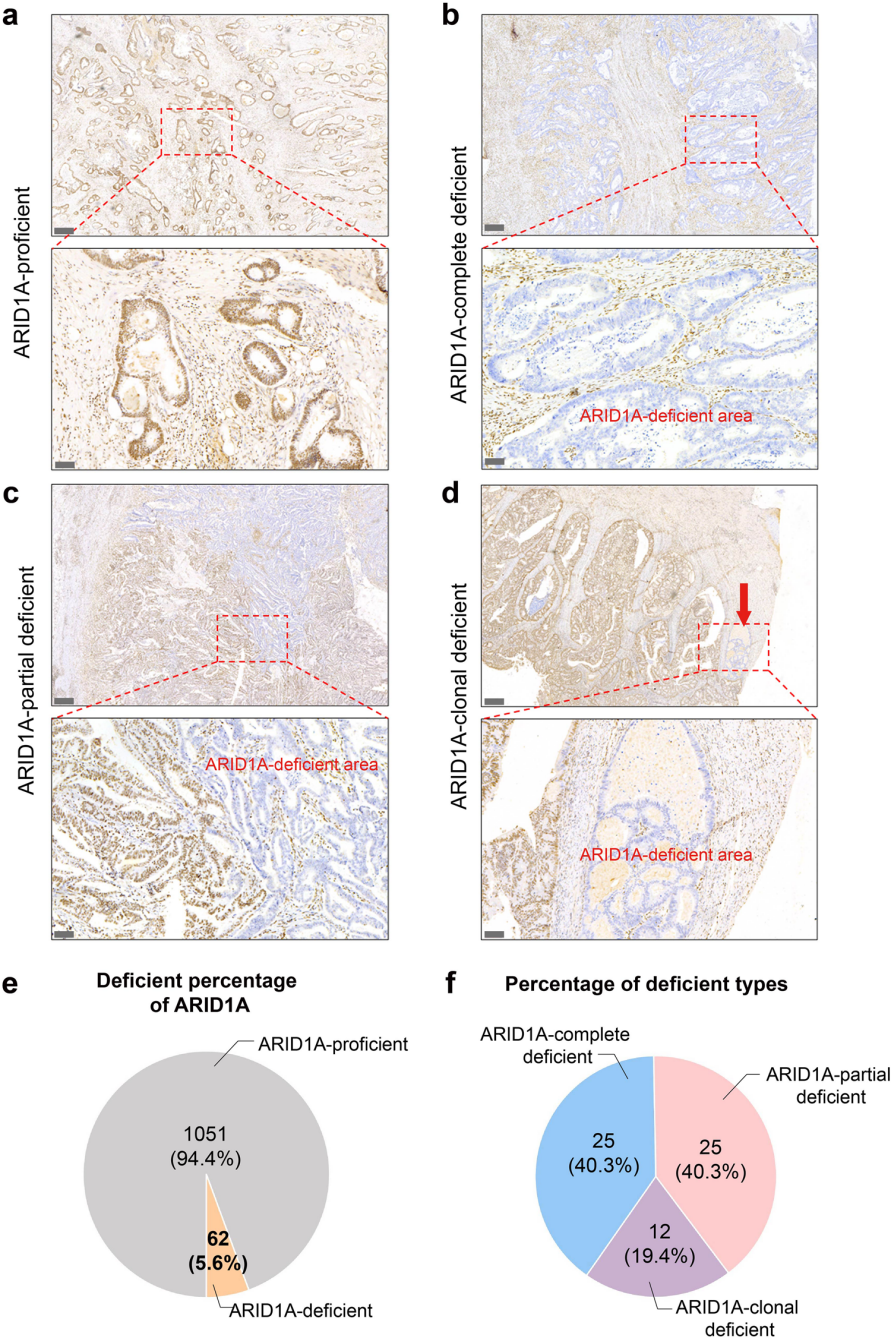
Proportion, classification and four patterns of cases with heterogeneous expression of ARID1A protein. Immunohistochemical detection showed ARID1A expression proficient (**a**), complete expression deficient (**b**), partial expression deficient (**c**), clonal expression deficient (**d**). Red fonts and red arrows mark ARID1A-deficient area. Scale bar for low magnification lens: 300 μm, scale bar for high magnification lens: 60 µm. **e** The proportion of ARID1A-deficient cases in total patients. **f** The proportion of three patterns cases with heterogeneous expression of ARID1A protein

To ensure the quality of the biomarkers, three pathologists from the Harbin Medical University Cancer Hospital analyzed digital images of stained tissue sections under 20 × magnification. ARID1A is usually localized in the nucleus. According to our research, ARID1A is expressed in different cell types in human colorectal cancer tissue, including epithelial cells, immune cells, and stromal cells. Epithelial cells with different ARID1A expression was classified as ARID1A-proficient (pARID1A), ARID1A-complete deficient (cdARID1A), ARID1A-partial deficient (pdARID1A), and ARID1A-clonal deficient (cldARID1A) (Fig. 1A). Image analysis software (Fiji/ImageJ) was used to estimate CD8 + , CD163 + , and FOXP3 + cell densities in tumor tissues, measured in cells/mm2, referring to our previous work [16].

### Statistical analysis

The clinicopathological features of patients were compared using statistical tests like the Wilcoxon-Mann–Whitney test or χ2 tests. These tests helped evaluate differences between various features. Kaplan–Meier survival analysis coupled with log-rank tests were employed. All statistical tests were two-sided, with a significance level set at P < 0.05, indicating statistical significance. The analysis was carried out using GraphPad Prism and SPSS.

## Result

### Heterogeneous expression patterns of ARID1A proteins in CRC samples

#### We detected the expression of ARID1A protein in 1113 CRC cases by IHC

There were 62 samples with heterogeneous expression of ARID1A proteins. Based on the distribution characteristics of ARID1A protein expression, there were four patterns of heterogeneous expression, including integrity expression (N = 1051, pARID1A), complete expression deficiency (N = 25, cdARID1A), partial expression deficiency (N = 25, pdARID1A), and clonal expression deficiency (N = 12, cldARID1A, Fig. 1a-d). For the pattern of complete expression deficiency (cdARID1A), ARID1A expression was absent in all tumor cells. For the pattern of partial expression deficiency (pdARID1A), the tumors with partial loss (≥ 20%) displayed intratumor heterogeneity in ARID1A staining. For the clonal expression deficiency (cldARID1A), tumors showed only focal, sporadic loss of ARID1A expression, confined to solitary glands. Statistical analysis revealed that the proportion of patients with ARID1A deficiency accounted for 5.6% of the total population (Fig. 1e). In further subgroup analysis, we observed a similar proportion of complete and partial deficiency patients in the ARID1A-deficient category (40.3%). Clonal expression deficiency was the least common type, constituting 19.4% of the total losses (Fig. 1f).

### Clinical and pathological characteristics of ARID1A-deficiency tumors

The correlation between ARID1A expression and clinicopathological characteristics was investigated in CRC patients. Table 1 provides an overview of clinicopathological data for the 1113 CRC patients, along with corresponding statistical outcomes. Among the ARID1A-proficient and ARID1A-deficient groups, significant disparities were identified in the MMR status (P value < 0.001). Table 2 delineates specific clinical-pathological characteristics related to distinct ARID1A deficiency types. Within the ARID1A deficient group, 40.3% exhibited ARID1A-complete deficiency, 40.3% had ARID1A-partial deficiency, and 19.4% were ARID1A-clonal deficient. Patients with complete and partial deletion of ARID1A showed a lower age of disease (average years 56.1 vs. 60.9, cdARID1A and pdARID1A group vs. cldARID1A group). Overall, no significant associations were found between the loss of ARID1A expression and any of the evaluated clinicopathological parameters.

**Table 1:** Clinical and pathological characteristics of 1113 colorectal cancer patients

	pARID1A (N = 1051)	dARID1A (N = 62)	P.value*
Age			0.178
≤ 60	560 (53.3%)	39 (62.9%)	
> 60	491 (46.7%)	23 (37.1%)	
Sex			0.322
Female	416 (39.6%)	29 (46.8%)	
Male	635 (60.4%)	33 (53.2%)	
Location			0.975
Left sided	196 (18.6%)	11 (17.7%)	
Right sided	327 (31.1%)	20 (32.3%)	
Rectum	528 (50.2%)	31 (50.0%)	
Grade			0.664
Well differentiation	84 (7.99%)	6 (9.68%)	
Moderate differentiation	913 (86.9%)	52 (83.9%)	
Poor-undifferentiation	54 (5.14%)	4 (6.45%)	
pT-stage			0.871
pT1	34 (3.24%)	1 (1.61%)	
pT2	109 (10.4%)	8 (12.9%)	
pT3	835 (79.4%)	49 (79.0%)	
pT4	73 (6.95%)	4 (6.45%)	
pN-stage			0.878
pN0	701 (66.7%)	41 (66.1%)	
pN1	249 (23.7%)	16 (25.8%)	
pN2	101 (9.61%)	5 (8.06%)	
TNM			0.977
I	115 (10.9%)	6 (9.68%)	
II	589 (56.0%)	35 (56.5%)	
III	339 (32.3%)	21 (33.9%)	
IV	8 (0.76%)	0 (0.00%)	
VELPI			0.876
Absent	951 (90.5%)	57 (91.9%)	
Present	100 (9.51%)	5 (8.06%)	
MMR status			< 0.001
dMMR	80 (7.61%)	20 (32.3%)	
pMMR	971 (92.4%)	42 (67.7%)	

*pARID1A* ARID1A-proficient, *dARID1A* ARID1A-deficient, *VELPI* Vascular invasion, *MMR* Mismatch repair, *dMMR* mismatch repair deficient, *pMMR* mismatch repair proficient

^*^χ2 test P-value

**Table 2:** Clinical and pathological characteristics of 1113 patients with ARID1A-deficiency tumors patients

	cdARID1A (N = 25)	pdARID1A (N = 25)	cldARID1A (N = 12)	P.value*
Age				0.054
≤ 60	14 (56.0%)	20 (80.0%)	5 (41.7%)	
> 60	11 (44.0%)	5 (20.0%)	7 (58.3%)	
Sex				0.242
Female	13 (52.0%)	13 (52.0%)	3 (25.0%)	
Male	12 (48.0%)	12 (48.0%)	9 (75.0%)	
Location				0.189
Left sided	7 (28.0%)	1 (4.00%)	3 (25.0%)	
Right sided	7 (28.0%)	10 (40.0%)	3 (25.0%)	
Rectum	11 (44.0%)	14 (56.0%)	6 (50.0%)	
Grade				0.726
Well differentiation	3 (12.0%)	1 (4.00%)	2 (16.7%)	
Moderate differentiation	20 (80.0%)	22 (88.0%)	10 (83.3%)	
Poor-undifferentiation	2 (8.00%)	2 (8.00%)	0 (0.00%)	
pT-stage				0.547
pT1	1 (4.00%)	0 (0.00%)	0 (0.00%)	
pT2	2 (8.00%)	3 (12.0%)	3 (25.0%)	
pT3	21 (84.0%)	19 (76.0%)	9 (75.0%)	
pT4	1 (4.00%)	3 (12.0%)	0 (0.00%)	
pN-stage				0.757
pN0	15 (60.0%)	17 (68.0%)	9 (75.0%)	
pN1	8 (32.0%)	5 (20.0%)	3 (25.0%)	
pN2	2 (8.00%)	3 (12.0%)	0 (0.00%)	
TNM				0.628
I	1 (4.00%)	3 (12.0%)	1 (8.33%)	
II	14 (56.0%)	9 (36.0%)	6 (50.0%)	
III	10 (40.0%)	13 (52.0%)	5 (41.7%)	
VELPI				0.833
Absent	24 (96.0%)	22 (88.0%)	11 (91.7%)	
Present	1 (4.00%)	3 (12.0%)	1 (8.33%)	
MMR status				0.636
pMMR	15 (60.0%)	18 (72.0%)	9 (75.0%)	
dMMR	10 (40.0%)	7 (28.0%)	3 (25.0%)	

*cdARID1A* ARID1A-complete deficient, *pdARID1A* ARID1A-partial deficient, *cldARID1A* ARID1A-clonal deficient, *VELPI* Vascular invasion, *MMR* Mismatch repair, *dMMR* mismatch repair deficient, *pMMR* mismatch repair proficient

^*^χ2 test P-value

### Abundance of tumor infiltrating lymphocytes of CD8 + , CD163 + , and FOXP3 + in heterogeneous expression of ARID1A samples

**Fig. 2:**
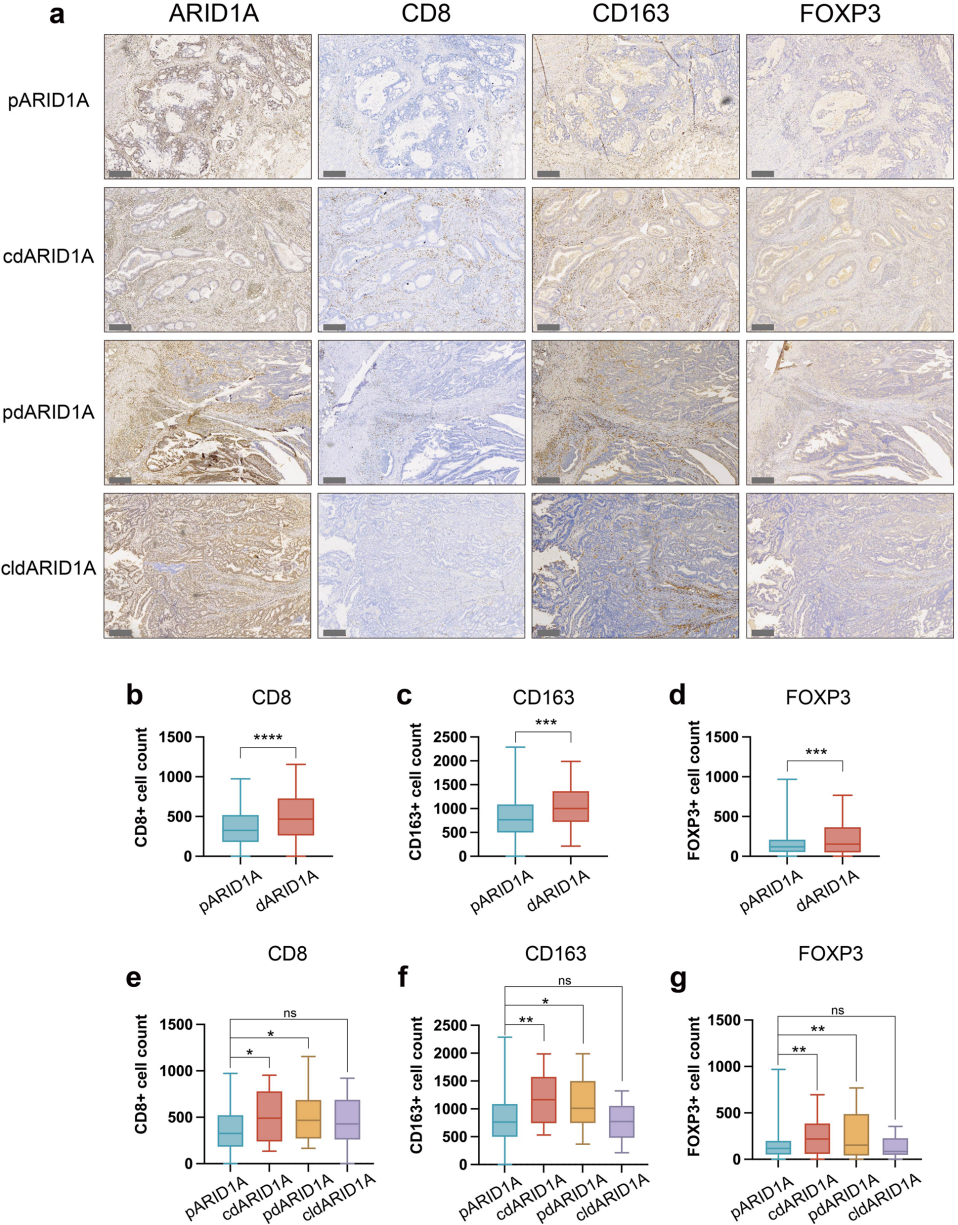
The abundance of tumor infiltrating lymphocytes in colorectal cancer with different ARID1A expression patterns. **a** The four cases with different ARID1A expression patterns and the corresponding representative images of CD8, CD163 and FOXP3 staining. Scale bar: 300 μm. The abundance of tumor infiltrating lymphocytes was compared between ARID1A-deficient and ARID1A-proficient patients, including CD8 + cells (**b**), CD163 + cells (**c**), and FOXP3 + cells (**d**). The abundance of CD8 + cells (**e**), CD163 + cells (**f**), and FOXP3 + cells (**g**) was compared in the four expression patterns of ARID1A. Statistical significance is indicated (*p < 0.05, **p < 0.01, ***p < 0.001, ****p < 0.0001)

**Fig. 3:**
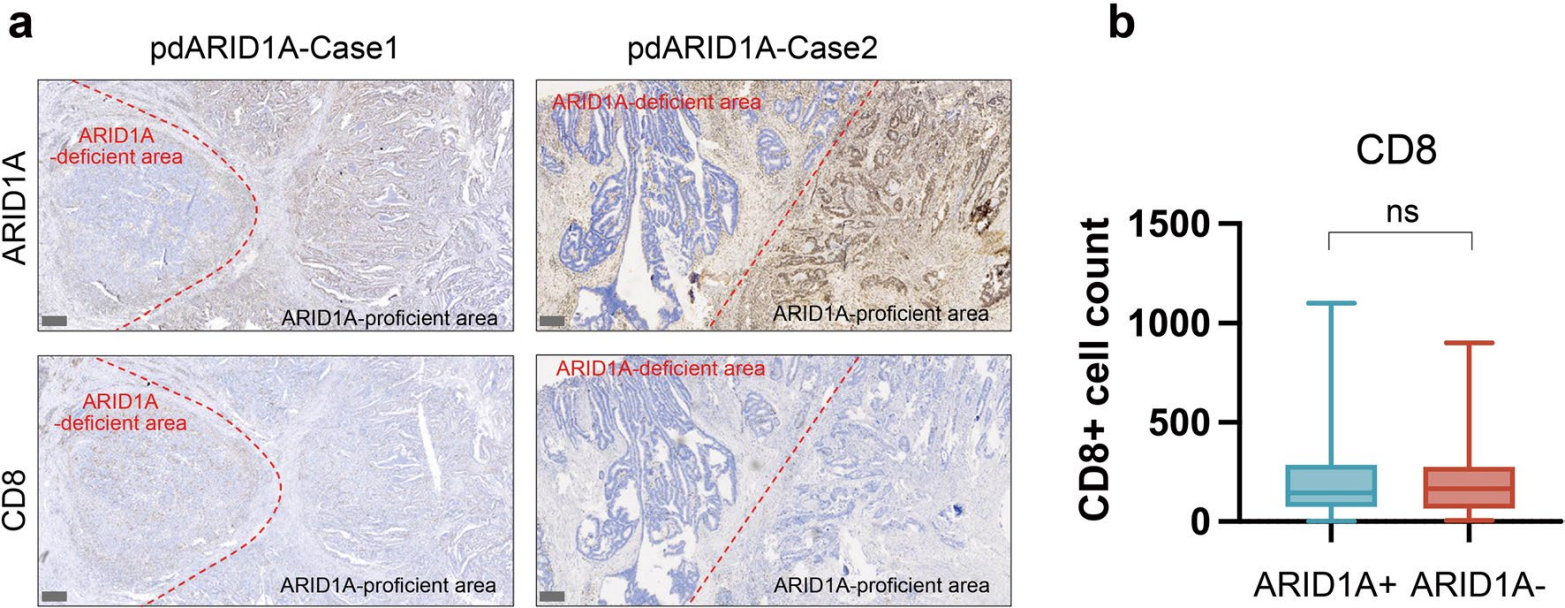
Comparison of CD8 + cells in ARID1A deletion region and ARID1A intact region in the CRC tissues with ARID1A partial expression deficient. **a** Division of ARID1A deletion region and intact region in CRC tissues. Scale bar: 300 μm. **b** The abundance of CD8 + cells between ARID1A deletion region and intact region

Next, we explored the relationship between the heterogeneous expression of ARID1A and the tumor lymphocyte infiltration. We examined the infiltration of various lymphocytes in tumors of CRC tissue utilizing IHC. In Fig. 2a, we assessed key lymphocyte subtypes, including CD8 + cells (marker of cytotoxic T cell), CD163 + cells (marker of macrophages), and FOXP3 + cells (marker of regulatory T cells), by integrating histological features such as cellular size and morphology. In comparison to ARID1A -proficient patients, patients with ARID1A deficiency exhibited a significant increase in the infiltration levels of CD8 + cells, CD163 + cells and FOXP3 + cells within the tumor tissue (Fig. 2b-d). However, in different subgroups, only samples with complete or partial deficiency of ARID1A showed a higher abundance of lymphocyte infiltration. In patients with ARID1A-clonal expression deficiency tumor, the infiltration patterns of these three immune cell types were comparable to those in ARID1A-proficient patients (Fig. 2e–g). Of note, regional comparisons within partial loss tumors showed comparable CD8 + cell densities between ARID1A null and ARID1A expressing areas (Fig. 3a, b). Together, these data indicate that heterogeneous expression of ARID1A reflects different abundance of tumor infiltrating lymphocytes.

### Heterogeneous expression of ARID1A protein is related to the prognosis and the efficacy of immunotherapy in CRC patients

**Fig. 4:**
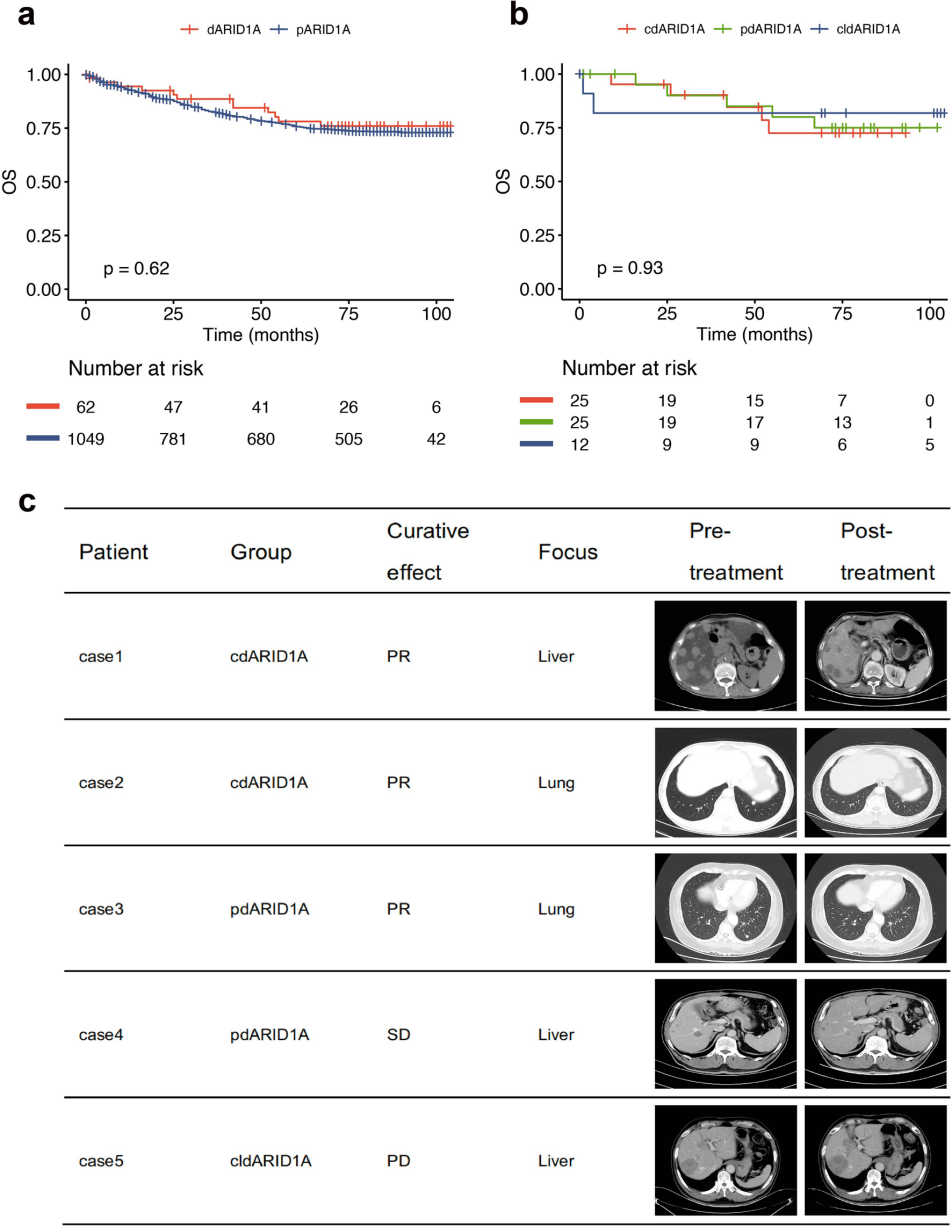
Heterogeneous expression of ARID1A protein is related to the prognosis and the efficacy of immunotherapy in CRC patients. **a** Kaplan–Meier survival curves of ARID1A-deficient and ARID1A-proficient patients. **b** Kaplan–Meier survival curves of CRC patients with three patterns of ARID1A-deficient. **c** Representative images of patients with ARID1A- deficient benefiting from anti-PD1 therapy or failing to benefit from anti-PD1 therapy. Scale bar: 100 µm

We further assessed associations between ARID1A status and overall survival in CRC patients. Kaplan–Meier survival analysis revealed a better overall survival in patients with ARID1A deficiency compared to those with ARID1A expression (Fig. 4a). Subsequently, we stratified patients based on the pattern of ARID1A loss. Patients with complete and partial ARID1A deficiency exhibited a trend towards prolonged survival compared to those in the ARID1A clonal expression deficiency group (Fig. 4b). Though underpowered for statistical significance due to limited cohort size, these data hint at a potential correlation between overall survival and extent of ARID1A loss. Moreover, we noted that ARID1A loss may increase immunotherapy sensitivity in CRC patients. Specifically, one patient with complete deficiency of ARID1A, confirmed by pathological analysis, achieved a partial response (PR) after only 3 cycles of anti-PD-1 therapy (Fig. 4c). In contrast, a patient with ARID1A clonal expression deficiency displayed progressive disease (PD) after an equivalent course of therapy (Fig. 4d). Though preliminary, these observations show that different expression patterns of ARID1A indicate differences in the efficacy of immunotherapy.

## Discussion

In our current study, we found that there were 62 cases of ARID1A-deficiency samples in 1113.

CRCs, which was close to the previously reported rate. Furthermore, our findings revealed heterogeneity in ARID1A, including complete expression deficiency, partial expression deficiency, and clonal expression deficiency. Tumors with loss of ARID1A expression do have an activating immune response. However, the heterogeneous expression of ARID1A is accompanied by a different immune landscape. The samples with clonal expression deficiency were similar to those with intact ARID1A expression, and the tumor infiltrating lymphocytes did not increase significantly. We also analyzed the samples with partial expression deficiency of ARID1A, and found that there was no significant difference in the abundance of CD8 + lymphocytes between ARID1A-loss region and expression region. Ultimately, our clinical observations demonstrated that CRC patients with ARID1A-clonal expression deficiency do not benefit from the treatment of ICIs. This result may help explain why patients with ARID1A mutations did not benefit from ICIs treatment.

Chou A.et al. has reported the heterogeneous expression of ARID1A in CRC [15]. The researchers examined the expression of ARID1A in 1876 CRCs from the TMA sections. A total of 110 (5.9% of all) CRCs demonstrated loss of staining for ARID1A, 105 cases were complete expression deficiency and 5 cases showed partial expression deficiency. The ratio of partial expression deficiency-samples in our results is significantly higher than that in this study, which we believe is due to the detection of TMA sections used in their study. Tissue microarray can only detect protein expression in very small tumor areas, which is difficult to capture partial expression deficiency, and clonal expression deficiency samples.

To the best of our knowledge, this is the first time to compare and analyze the immune landscape between heterogeneously expressed ARID1A samples. In our results, we found that the samples with complete and partial expression deficiency exhibited similar active tumor immune response, but the samples with loss of clonal expression were completely different. This phenomenon may affect the accuracy of ARID1A in predicting the efficacy of immunotherapy for CRC. Except for CRC patients with defective mismatch repair/microsatellite instability-high (dMMR/MSI-H) and POLD1/POLE mutations, there are still patients who are expected to benefit from ICIs treatment. Despite the heterogeneous expression, ARID1A is still a promising marker for predicting the efficacy of immunotherapy, especially for screening potential beneficiaries from patients with MSS-CRC [17–23]. To improve the accuracy of the marker, the strategy of marker combination can be considered. A study revealed that the combination of ARID1A plus CXCL13 may improve prediction capability for metastatic urothelial carcinoma receiving ICIs [24, 25].

In conclusion, our study revealed the expression of ARID1A in CRC and its relationship with immune landscape through IHC detection of postoperative CRC specimens. It deepens the understanding of the effect of ARID1A on the immune response of CRC and explains the potential reason why some CRC patients with ARID1A mutation cannot benefit from immunotherapy [26]. IHC, rather than next generation sequencing (NGS), can more accurately determine the role of ARID1A in predicting the effect of immunotherapy. This will help to better promote the use of ARID1A to guide clinical practice.

## Data Availability

The data used to support the findings of this study are available from the corresponding author upon request.

## Abbreviations

ARID1A: AT-rich interaction domain 1A
SWI/SNF: Switch/Sucrose Non-Fermentable
CRC: Colorectal cancer
DSBs: DNA double-strand breaks
DDR: DNA damage repair
PI3K: Phosphatidylinositol 3-kinase
PKB: Protein kinase B
MSS: Microsatellite stability
pMMR: Proficient mismatch repair
ACT: Adjuvant chemotherapy
CT: Central tumor
IM: Invasive margin
TIMs: Tumor-infiltrating myeloid cells
TMA: Tissue microarray
NGS: Next generation sequencing
